# Validation, analysis and annotation of cryo-EM structures

**DOI:** 10.1107/S2059798321006069

**Published:** 2021-08-31

**Authors:** Grigore Pintilie, Wah Chiu

**Affiliations:** a Department of Bioengineering, James H. Clark Center, Stanford University, Stanford, CA 94305, USA; b Division of Cryo-EM and Bioimaging, SSRL, SLAC National Accelerator Laboratory, Stanford University, Menlo Park, CA 94025, USA

**Keywords:** cryo-EM, validation, Fourier shell correlation, modeling, annotation

## Abstract

The process of turning 2D micrographs into 3D atomic models of the imaged macromolecules has been under rapid development and scrutiny in the field of cryo-EM. Here, some important methods for validation at several stages in this process are described. Firstly, how Fourier shell correlation of two independent maps and phase randomization beyond a certain frequency address the assessment of map resolution is reviewed. Techniques for local resolution estimation and map sharpening are also touched upon. The topic of validating models which are either built *de novo* or based on a known atomic structure fitted into a cryo-EM map is then approached. Map–model comparison using *Q*-scores and Fourier shell correlation plots is used to assure the agreement of the model with the observed map density. The importance of annotating the model with *B* factors to account for the resolvability of individual atoms in the map is illustrated. Finally, the timely topic of detecting and validating water molecules and metal ions in maps that have surpassed ∼2 Å resolution is described.

## Abstract 2

Methods for the validation of 3D maps from cryo-EM are described and illustrated using an example map of bacteriophage P22. Methods for fitting, flexible fitting, refinement and annotation of models generated based on such maps are also explored.

## 1. Introduction   

Cryo-EM is becoming more widely used to obtain 3D maps of biomedically important macromolecular complexes at increasing resolution. The process begins with image acquisition, in which 2D images or micrographs are obtained, typically as a stack of movie frames (Li *et al.*, 2013 ▸). The frames are averaged while applying motion correction, which adjusts for the motion of particles during image acquisition (Campbell *et al.*, 2012 ▸). From the motion-corrected images, particles are then picked, typically manually at first, and then automatically by template matching or convolutional neural networks trained on the manually picked images (Bell *et al.*, 2018 ▸; Bepler *et al.*, 2019 ▸; Wagner *et al.*, 2019 ▸).

The picked particles are then aligned in order to find 2D class averages, which in turn are used to generate an initial 3D model (Bell *et al.*, 2016 ▸; Punjani *et al.*, 2017 ▸; Zivanov *et al.*, 2018 ▸). Finally, the 3D model is iteratively refined. At each iteration, the likely orientation of each 2D particle with respect to this 3D model is found. The combination of all particles averaged together by weighted back-propagation or inverse Fourier transform yields more and more detailed 3D volumes (Tang *et al.*, 2007 ▸; Zivanov *et al.*, 2018 ▸). Since the biochemically purified particles do not necessarily assume a single conformation, particle-classification methods have been developed to identify similar subsets of particles yielding structures of different states (Punjani *et al.*, 2017 ▸; Zivanov *et al.*, 2018 ▸).

## 2. Pitfalls in reconstruction   

One pitfall in the reconstruction process is that particles may be small or hard to see due to the high noise that is typical in the micrographs. The introduction of direct detectors has allowed the use of movie frames and an increase in the signal-to-noise ratio after motion correction (Li *et al.*, 2013 ▸). Furthermore, improvements in particle-picking and denoising methods have made it easier to ensure that good particles are found in the micrographs (Bell *et al.*, 2018 ▸; Bepler *et al.*, 2019 ▸; Wagner *et al.*, 2019 ▸). Still, the ‘Einstein from noise’ effect could lead to the over-refinement of noise rather than signal (Henderson, 2013 ▸). More sophisticated reference-free initial model generation and probabilistic considerations during refinement (Punjani *et al.*, 2017 ▸; Zivanov *et al.*, 2018 ▸) are now more likely to produce correct 3D volumes.

Another possible pitfall in image reconstruction is that of preferred orientations. This happens if particles are not found in all possible orientations in the vitrified sample. Unless the particles have a high degree of symmetry, this typically results in a reconstruction in which features may not be resolved equally well in all directions. The latter is referred to as resolution anisotropy, and it can potentially be addressed by imaging the specimen at a tilt angle (Tan *et al.*, 2017 ▸). Analysis of images at small tilt angles can also help to prevent another pitfall: that of incorrect orientation determination of each particle (Henderson *et al.*, 2011 ▸). Incorrect orientations can result in incorrect 3D maps that may not look like the actual macromolecule. Furthermore, the accuracy with which particle orientations can be determined limits the resolution or resolvability of features in the reconstruction (Henderson *et al.*, 2011 ▸).

## 3. Gold-standard map-resolution estimation   

**Figure 1:**
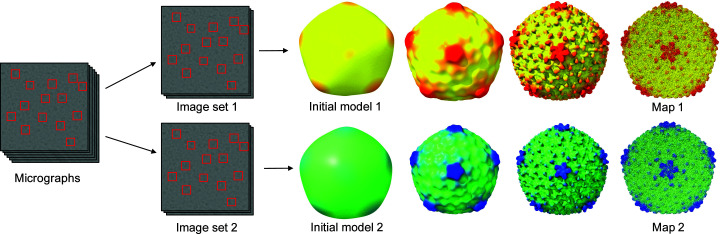
Map validation via independent reconstructions. The particle images are split into two separate sets and the reconstruction is performed separately for each set (Hryc *et al.*, 2017 ▸).

An important part of the 3D reconstruction process is to estimate the resolution of the final map, as this can inform about the amount of detail that should be visible in the map. The recommended way to estimate the resolution accurately is known as gold-standard resolution estimation (Henderson *et al.*, 2012 ▸). The procedure is to split the particle images into two sets and reconstruct a 3D volume from each set independently. Fig. 1 ▸ illustrates this process for a map of bacterophage P22 reconstructed to 3.3 Å resolution (Hryc *et al.*, 2017 ▸). This procedure is used in most modern cryo-EM reconstruction methods.

**Figure 2:**
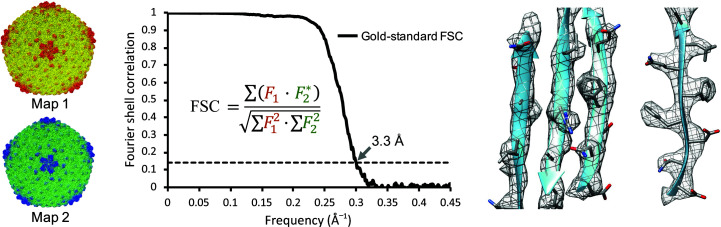
Fourier shell correlation (FSC) between two independent 3D reconstructions of bacteriophage P22 (EMDB entry EMD-8606). A section of the map with the model is shown on the right, showing the good separation of a β-sheet and visibility of bulky side chains which are expected at ∼3.5 Å resolution (Hryc *et al.*, 2017 ▸).

The map resolution is then estimated by Fourier shell correlation (FSC); the two independent reconstructions are converted into Fourier components and the correlation is calculated in shells, where each shell corresponds to a frequency or resolution (Frank & Al-Ali, 1975 ▸; Harauz & van Heel, 1986 ▸). Such an FSC plot is shown in Fig. 2 ▸. At low frequencies (low resolution) the FSC tends to be high (close to 1), meaning that the two independent maps agree. Towards higher frequencies (higher resolutions) the FSC eventually drops as the maps become less consistent. The resolution at which the FSC drops below 0.143 is widely considered to be an estimate of the resolution of the map (Rosenthal & Henderson, 2003 ▸), although the use of such an exact threshold is also often debated (van Heel & Schatz, 2005 ▸). This definition of resolution is different from the traditional definitions in crystallography or in optics. Our resolution refers to the level that map features from two independent reconstructions are consistent with each other within this resolving limit. As an example, an FSC plot for a map of bacteriophage P22 is shown in Fig. 2 ▸. The FSC drops below 0.143 at a resolution of 3.3 Å.

The resolution of the map should be indicative of the degree to which features are resolved (Chang *et al.*, 2012 ▸). For example, at ∼9 Å secondary structures such as α-helices and β-sheets become discernible and at ∼4 Å individual strands within β-sheets become separable and bulky side chains start to become visible (Pintilie & Chiu, 2018 ▸). Thus, observing that β-strands are indeed separable and side-chain densities are visible, as shown in Fig. 2 ▸, supports the estimated resolution of 3.3 Å for the P22 reconstruction.

## 4. High-resolution noise substitution/phase randomization   

Overfitting in image reconstruction arises primarily when the map being refined is iteratively fitted to each particle image so as to amplify noise rather than signal. A method to detect this is based on high-resolution noise substitution or phase randomization (Chen *et al.*, 2013 ▸). In both cases, the initial data set (particle images) is modified to substitute or randomize phases higher than a certain frequency, usually 75% of the resolution measured by FSC between the two data sets. The two independent reconstructions are then recalculated from the modified data set, and an FSC is plotted between the two resulting maps. Since phases should become non-correlated between the two reconstructions with either procedure beyond a certain frequency, any correlations beyond this frequency are likely to be due to overfitting.

**Figure 3:**
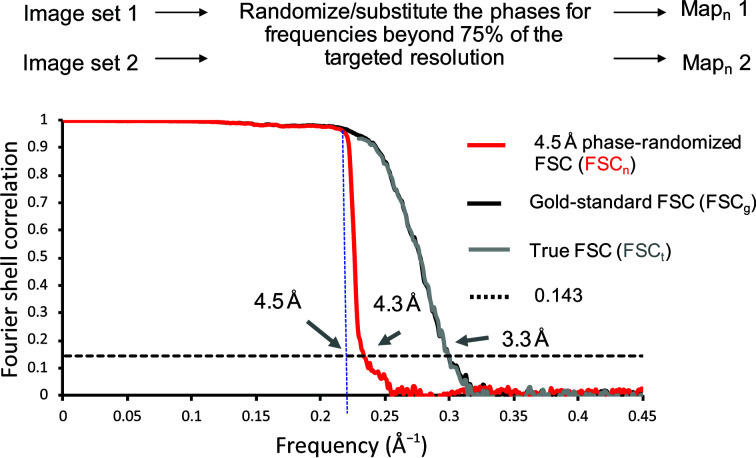
Use of phase randomization for map validation applied to P22 reconstruction (Hryc *et al.*, 2017 ▸). The FSC between independent reconstructions is plotted for the original data (FSC_g_) and also for the same data after phase randomization at frequencies higher than 4.5 Å (FSC_n_). The true FSC (FSC_t_) represents correlations above 4.5 Å which are likely to be due to genuine signal rather than overfitted noise.

Fig. 3 ▸ shows FSC plots with and without phase random­ization obtained for bacteriophage P22 data (Hryc *et al.*, 2017 ▸). The plot between the maps obtained with the original data set is labeled FSC_g_, while the plot obtained after phase random­ization is labeled FSC_n_. Phases beyond 4.5 Å, which is 75% of the target resolution of 3.3 Å, were randomized. In FSC_n_, the correlation drops dramatically beyond this resolution of 4.5 Å, indicating that over-refinement does not occur. The true FSC, or FSC_t_, is calculated from FSC_g_ and FSC_n_, and is meant to represent the correlation between genuine structural features rather than overfitted noise. In Fig. 3 ▸, the plot shows that FSC_t_ is very close to FSC_g_ and also drops below 0.143 at 3.3 Å.

## 5. Local resolution   

The resolvability of atomic features can vary throughout a map. For example, parts of the macromolecule may be more flexible than others and have a different conformation in each imaged particle (Herzik *et al.*, 2019 ▸). Such components would lead to lower resolvability, as the average of all of the particles would be more diffuse than if the same part were the same in each particle. Another possibility is that some residues may be more prone to radiation damage (Hattne *et al.*, 2018 ▸). The side chains of such residues may appear to be less resolved, or not visible to the same degree as other residues which are better resolved in the same map (Barad *et al.*, 2015 ▸). Evaluation of local resolution can be performed using methods such as *Bsoft* (Heymann & Belnap, 2007 ▸), *ResMap* (Kucukelbir *et al.*, 2014 ▸) or *MonoRes* (Vilas *et al.*, 2018 ▸), which can point out areas such as these where the model may be incomplete or less certain.

## 6. Map sharpening   

Post-processing of the map is an important step to allow proper interpretation of the final 3D map in real space. While phases are well represented in the real-space 2D images, amplitudes tend to be attenuated and hence incorrect at higher frequencies due to the contrast-transfer and envelope functions applied to each micrograph and particle; in contrast, in X-ray crystallography the amplitude scale is well determined, whereas the phases are not (Cheng, 2015 ▸). Thus, the reconstructed cryo-EM map may appear smoother in real space than it should if the amplitude weights were correct. Post-processing of cryo-EM maps tries to correct them by re-weighting higher frequencies in Fourier space, a process often referred to as sharpening (Rosenthal & Henderson, 2003 ▸). This process can be performed either globally or locally, with the latter involving a different filter at each point in the map (Jakobi *et al.*, 2017 ▸; Ramírez-Aportela *et al.*, 2020 ▸; Terwilliger, Sobolev *et al.*, 2018 ▸). Such sharpening procedures bring out detail in the structure that may otherwise not be visible in real space, allowing more accurate models to be created. However, it is also possible that if taken too far, noise may be amplified excessively as a result. The latter results in 3D maps with disconnected densities that do not look like proper atomic models (Pintilie *et al.*, 2020 ▸).

## 7. Model building and fitting   

At resolutions higher than ∼3.5 Å it is often possible to create polypeptide backbone models *de novo* in the reconstructed map, for example interactively using *Coot* (Emsley *et al.*, 2010 ▸) or using automated methods (Baker *et al.*, 2012 ▸; Cowtan, 2006 ▸; Terwilliger, Adams *et al.*, 2018 ▸). Since resolution can vary throughout the maps, complete models may not be possible. At lower resolutions, known atomic structures of the components or domains can be fitted to the map. This is usually performed using a ‘rigid fit’, where the model is simply translated and rotated as a rigid body, varying only six degrees of freedom (Birmanns *et al.*, 2011 ▸; Roseman, 2000 ▸). The map can also be segmented first to help to identify individual components (Pintilie *et al.*, 2010 ▸). Moreover, confidence levels in rigid fitting can be based on statistical analyses using *Z*-scores (Pintilie & Chiu, 2012 ▸). Rigid fitting is often followed by further flexible fitting (Joseph *et al.*, 2016 ▸; Trabuco *et al.*, 2008 ▸) or refinement (Afonine, Poon *et al.*, 2018 ▸; Brown *et al.*, 2015 ▸), where atoms are moved individually so as to better match the observed density.

Restraints are typically used to prevent overfitting during flexible fitting or refinement. At higher resolutions (for example 3.5 Å and better) only basic restraints are typically needed, for example bond lengths, angles and dihedrals, as there is sufficient information in the map to resolve the backbone geometry, side-chain rotamers and secondary structures. At lower resolutions, further restraints are typically used to maintain proper geometry, for example secondary-structure restraints (for example via hydrogen bonds), restraints for backbone dihedrals and side-chain rotamers, and restraints to prevent clashing of nearby atoms. Higher-level restraints can also be used to maintain overall fold/domain structure at lower resolutions, for example via elastic networks (Schröder *et al.*, 2007 ▸) or ‘jelly bodies’ (Murshudov *et al.*, 2011 ▸). Instead of restraints, parts of the model can also be kept rigid, for example in *FlexEM* (Joseph *et al.*, 2016 ▸). Restraints/rigidity can be applied at local levels to reflect the local resolution, and tools typically allow this through user-defined constraints/rigidity which can be varied in different parts of the model.

## 8. Model-to-map fit   

A model fitted or built into a map can be scored on how well it matches the map density in several ways. Amongst the first metrics to be introduced were atom inclusion and average density at atom positions (Lagerstedt *et al.*, 2013 ▸; Rossmann *et al.*, 2001 ▸). More recent scores calculate a cross-correlation between the cryo-EM map and a model map (Joseph *et al.*, 2017 ▸; Pintilie & Chiu, 2012 ▸; Roseman, 2000 ▸), or using difference maps (Joseph *et al.*, 2020 ▸). The model map can be calculated by blurring the atom coordinates using a Gaussian filter, for example using the *molmap* command in *UCSF Chimera* (Goddard *et al.*, 2007 ▸), or using electron scattering factors for each atom, for example using *phenix.fmap* or *REFMAC* (Afonine, Poon *et al.*, 2018 ▸; Nicholls *et al.*, 2018 ▸). The model map can be generated at any desired resolution, although usually it is chosen to be similar to that of the cryo-EM map. Another metric similar to cross-correlation which can be calculated for each atom individually is the *Q*-score, which will be described further in Section 10. Various metrics were compared in the recent EMDR-organized ‘2019 Model Metrics Challenge’ (Lawson *et al.*, 2021 ▸).

One useful way to evaluate the map-to-model match is to use an FSC plot between the cryo-EM map and a simulated map of the fitted/built model (Afonine, Klaholz *et al.*, 2018 ▸; Brown *et al.*, 2015 ▸; Rosenthal & Rubinstein, 2015 ▸). The model map should be generated at a similar or higher resolution to the cryo-EM map and, because it contains no noise, the map–model FSC should cross the 0.5 threshold at a similar resolution as the two independent half-maps (Rosenthal & Henderson, 2003 ▸). Such a plot informs on how well atomic features are resolved in the map. This is because the FSC plot shows correlations at each resolution, so the higher the resolution at which the cryo-EM map and the model agree, the more resolved the features in the map are expected to be. One drawback of this method is that it applies to the entire map/model. Masking small regions/features (for example a single side chain) is impractical as the mask boundary which has to be applied to both the cryo-EM map and the model map tends to introduce artificial correlations (Pintilie *et al.*, 2016 ▸).

## 9. Flexible fitting and refinement: detection of overfitting   

**Figure 4:**
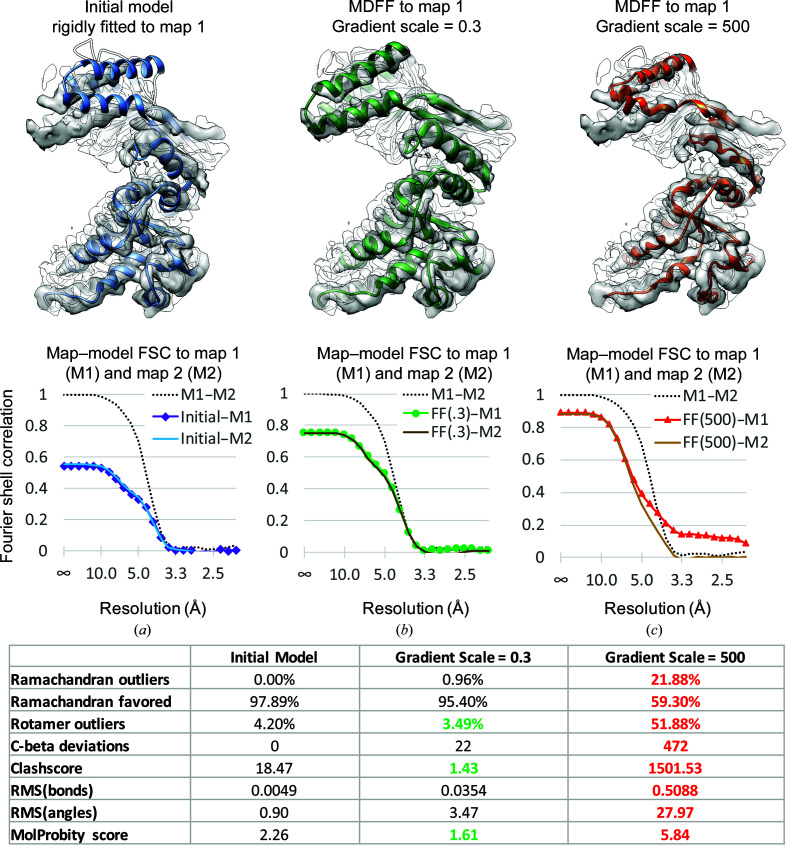
Test of model overfitting in a map of GroEL at 4.1 Å resolution (EMDB entry EMD-6422) with PDB entry 4ki8. The model was rigidly fitted to the map (*a*) and then flexibility fitted with MDFF using gradient weights of 0.3 (*b*) and 500 (*c*). Map–model FSC plots are shown below each model, along with gold-standard FSC plots between the independent maps M1 and M2. At the bottom, model-only scores for each model are tabulated.

To test for the overfitting of a model due to noise, a commonly suggested procedure is to flexibly fit or refine the model into one of the independent maps produced during the reconstruction (map 1) and test the model in the other independent map (map 2), which should have noise independent of the latter (Brown *et al.*, 2015 ▸; DiMaio *et al.*, 2013 ▸; Pintilie *et al.*, 2016 ▸). Fig. 4 ▸ illustrates the results of such a process, with FSC plots of the model versus the map determined at moderate resolution (∼4 Å). Three plots are shown: the initial model (rigidly fitted X-ray structure) versus cryo-EM maps 1 and 2 (Fig. 4 ▸ *a*), the model after molecular-dynamics flexible fitting (MDFF) with a gradient scale of 0.3 (the recommended value; Fig. 4 ▸ *b*) and the model after MDFF with a gradient scale of 500 (pushing on atoms much more forcefully in the direction of the density gradient; Fig. 4 ▸ *c*). The units of the gradient scale variable are kcal mol^−1^, and the recommended value of 0.3 results in forces of the order of 10–15 pN (Trabuco *et al.*, 2008 ▸).

In Fig. 4 ▸(*b*), the FSC plots show that the model better fits the map (higher FSC), but the FSC for the model against maps 1 and 2 stay within the FSC of map 1 to map 2, *i.e.* the model has not been overfitted to noise. On the other hand, in Fig. 4 ▸(*c*) the FSC for the model to map 1 has now gone beyond the map 1–map 2 FSC at high frequencies, *i.e.* the model has been overfitted to noise. Geometry scores for the three models calculated with *phenix.molprobity* (Chen *et al.*, 2010 ▸) are also shown in Fig. 4 ▸. The scores for the model fitted using a gradient scale of 0.3 are close to those of the initial model, with the clashscore and overall *MolProbity* score actually being improved; however, for the model fitted using a gradient scale of 500 they are dramatically worse, showing that the model geometry was compromised as a result of overfitting to the cryo-EM map.

## 10. Model-to-map fit: *Q*-score   

The *Q*-score is a type of cross-correlation score that is applied to each individual atom to measure how well it is resolved (Pintilie *et al.*, 2020 ▸). The calculation involves comparing values around each atom, at increasing radial distances, with values taken from a reference Gaussian. The parameters that define the reference Gaussian include the height and width. The height is based on the range of values in the map. The width was initially set to 0.6 Å, which made the reference Gaussian match a well resolved atom at ∼1.4 Å resolution. Since then, maps have been obtained at up to 1.15 Å resolution; hence the width here is adjusted to 0.4 Å, such that the highest *Q*-score is obtained for a well resolved atom at ∼1 Å resolution.

**Figure 5:**
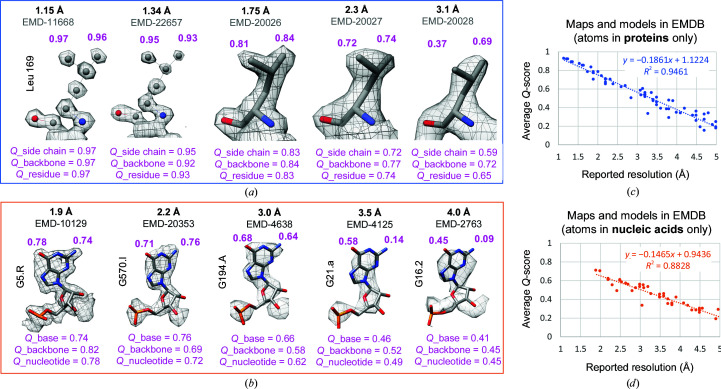
*Q*-scores for atoms in protein residues (*a*) and nucleotides (*b*) from cryo-EM maps at different resolutions. Average *Q*-scores for entire models and maps in the EMDB are plotted for atoms in protein residues (*c*) and in nucleotides (*d*) versus the reported resolution; each point represents a map and the model deposited with the map. On average, *Q*-scores correlate very well with the reported resolutions of the maps.

Fig. 5 ▸ illustrates *Q*-scores for atoms in proteins and nucleic acids at different resolutions. The highest *Q*-scores are obtained for atoms in the map of apoferritin at 1.15 Å (Fig. 5 ▸ *a*). As the resolvability of the atom decreases, so does the *Q*-score. *Q*-scores can also be averaged to represent the resolvability of an entire residue or nucleotide, as shown in Figs. 5 ▸(*a*) and 5 ▸(*b*). Figs. 5 ▸(*c*) and 5 ▸(*d*) show plots of *Q*-scores averaged over entire models and maps as deposited in the EMDB (Lawson *et al.*, 2011 ▸) versus reported resolutions. A strong correlation can be seen in both cases. This indicates that the *Q*-score is a good indicator of the resolvability of atomic features in cryo-EM maps, much as the FSC-estimated resolution typically is.

**Figure 6:**
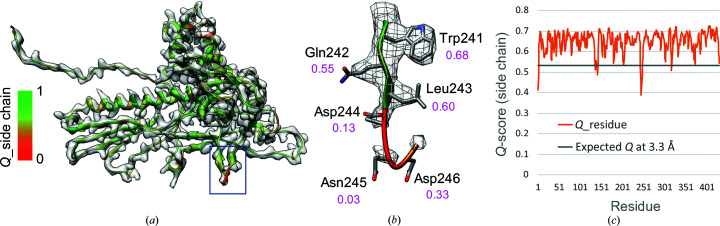
Per-residue *Q*-scores of P22 coat protein (EMDB entry EMD-8606, PDB entry 5uu5). In (*a*), the side-chain *Q*-score is color-coded on the ribbon display. (*b*) shows a few residues up close, some of which are well resolved and have a high *Q*-score (*e.g.* Trp241) and some of which are not resolved and have a low *Q*-­score (*e.g.* Asn245). In (*c*), the side-chain *Q*-score is plotted for each residue. Most residues are well resolved and have *Q*-scores above the expected *Q*-score at the estimated resolution of the map. The expected *Q*-score is calculated from the formula in Fig. 5 ▸, in this case using the plot considering protein atoms.

Per-residue *Q*-scores can also be used to visualize resolvability on a ribbon model of a protein, as shown in Fig. 6 ▸. The protein comes from an icosahedral reconstruction of the P22 virion (Hryc *et al.*, 2017 ▸). Such depictions can be very useful as an inspection or validation/reporting tool. When creating plots of per-residue plots, as in Fig. 6 ▸(*c*), the residue *Q*-scores can be compared with the expected *Q*-score at the resolution of the map, shown as a horizontal gray bar. The expected *Q*-score can be calculated given the resolution of a map using the formulas shown in Figs. 5 ▸(*c*) and 5 ▸(*d*) for proteins or nucleic acids, respectively. When the *Q*-scores are above the expected *Q*-score, one can have more confidence that those residues are both modeled properly and resolved in the map as expected for the estimated resolution of the map. On the other hand, when the *Q*-scores dip below the expected *Q*-score, this could indicate that those residues are not resolved or fitted properly to the map. This would then be confirmed visually: if the residues indeed appear not to be fitted properly, manual adjustment or automated refinement could be used to improve the fit.

## 11. Atomic *B* factors   

In X-ray crystallography, *B* factors or atomic displacement parameters (ADPs) are often used to represent the degree of freedom of movement of each atom and are refined directly from the diffraction data (Murshudov *et al.*, 1999 ▸). In cryo-EM-derived models, *B* factors/ADPs can also be calculated by first converting the real-space cryo-EM map to structure factors and applying the same techniques, for example with *REFMAC* or *Phenix*. Restraints are typically applied, so that bonded atoms affect each other’s *B* factor/ADP. Using *phenix.real_space-refine*, per-residue *B* factors/ADPs can also be calculated directly in real space.

Cryo-EM map-derived models are often deposited without consideration of *B* factors or their meaning (Wlodawer *et al.*, 2017 ▸), although efforts towards accurate model annotation with ADPs (equivalent to *B* factors) have previously been made (Hryc *et al.*, 2017 ▸). When simulating a map from a model, for example with *phenix.fmap*, *B* factors are used to modulate the scattering factors applied to each atom (Afonine, Klaholz *et al.*, 2018 ▸). When the *B* factors are modulated properly, the model-derived map should match well with the cryo-EM map.

**Figure 7:**
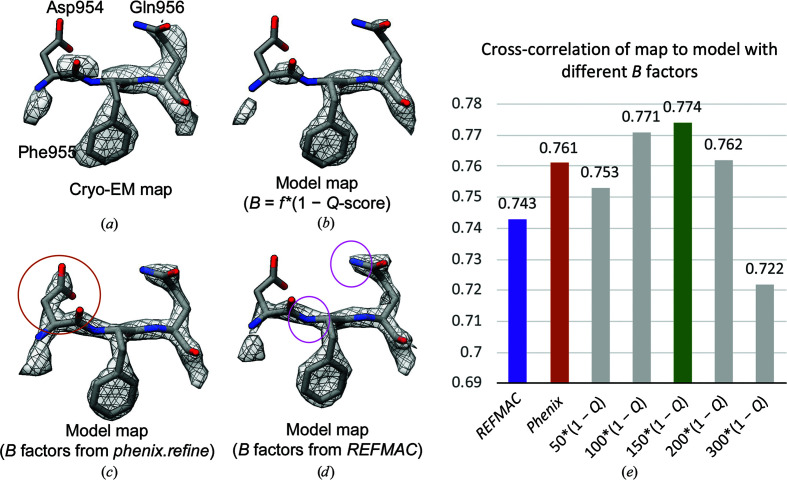
Model and extracted density around three residues in a 2.2 Å resolution cryo-EM map of β-galactosidase (EMDB entry EMD-2984, PDB entry 5a1a). The cryo-EM map is shown in (*a*) and maps generated from models with *B* factors with different methods in (*b*)–(*d*). (*e*) shows real-space cross-correlations between the entire cryo-EM map and model maps with *B* factors calculated using *REFMAC*, *Phenix* and *Q*-scores.

*Q*-scores are related to the density spread around each atom, and thus also potentially to *B* factors. Hence, they were also proposed as a potential way to calculate *B* factors via the formula *B* = f*(1 − *Q*) (Zhang *et al.*, 2020 ▸). In Fig. 7 ▸, three ways of generating *B* factors are compared: *Q*-scores (with several values of *f*), *phenix.refine* and *REFMAC*. Three residues and the extracted cryo-EM density around them are shown in Fig. 7 ▸(*a*). Figs. 7 ▸(*b*), 7 ▸(*c*) and 7 ▸(*d*) show the model maps generated using *B* factors from each of the three methods. The maps were generated using *phenix.fmap*, inputting the model with *B* factors generated by each method, at a resolution of 2.2 Å as reported for the cryo-EM map.

For the residues in Fig. 7 ▸, *B* factors based on *Q*-scores reproduce the cryo-EM maps better than the other two methods. For example, the Asp954 residue is not resolved when using *B* factors based on *Q*-scores, as in the cryo-EM map, whereas it appears resolved when using *Phenix* *B* factors. With *B* factors from *Q*-scores, the N atom in the Phe955 backbone and the atom N in the Gln956 side chain appear unresolved, again matching the cryo-EM map, whereas they appear resolved when using *Phenix*- and *REFMAC*-calculated *B* factors.

Fig. 7 ▸(*e*) plots the cross-correlation (CC) score between the simulated maps of the entire complex and the cryo-EM map. The CC score is highest when using *B* factors based on *Q*-scores with *f* = 150. To determine the optimal *f*, several values of *f* were tried as shown in the plot in Fig. 7 ▸(*e*), including *f* = 50, 100, 150, 200 or 300. The CC score was highest for *f* = 150; hence, this value was determined to produce the best *B* factors in this case. The value of *f* that produces the optimal *B* factors for a model and map may depend on the resolution of the map, because *Q*-scores inherently relate to both the resolution of the map and also the spread of density around a given atom due to its dynamical properties. Thus, it is very likely that different values of *f* as a scaling parameter would be needed in different maps. Regardless, the value of *f* could be determined independently for each map and model without other information, as performed here.

## 12. Water molecules and ions   

As cryo-EM maps have reached resolutions beyond 3 Å, water molecules and other metal ions can now start to be observed around protein and RNA molecules. Water molecules and ions are often placed in X-ray maps, for example with tools such as *Phenix* (Echols *et al.*, 2014 ▸), *Coot* (Emsley *et al.*, 2010 ▸) and *ARP*/*wARP* (Langer *et al.*, 2013 ▸). Such methods have also been applied to cryo-EM maps; however, they consider only water molecules and do not yet attempt to differentiate between water molecules and ions.

Another recently proposed method, SWIM (segmentation-guided water and ion modeling), attempts to place both water molecules and ions in cryo-EM maps (Zhang *et al.*, 2020 ▸), differentiating between them based on criteria outlined in the *UnDowser* procedure (Prisant *et al.*, 2020 ▸). These criteria consider distances between the water molecules/ions and nearby protein/nucleic atoms. For example, an ion tends to be closer to such atoms, and it would be considered to clash if it was modeled as a water molecule.

**Figure 8:**
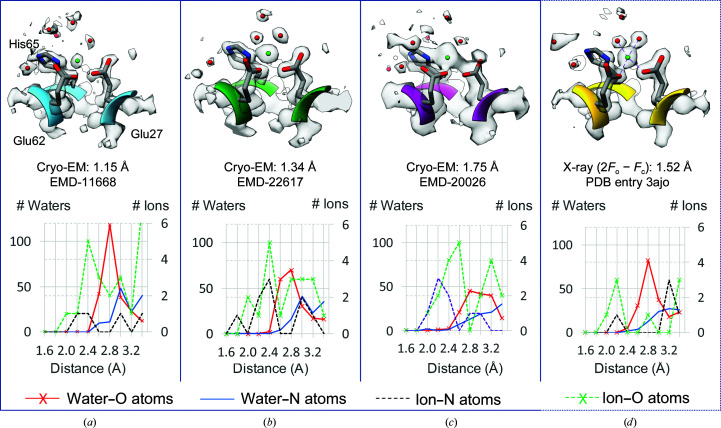
An ion (green ball) and several water molecules (red balls) in the vicinity of three residues from apoferritin. (*a*), (*b*) and (*c*) show cryo-EM maps and models, while (*d*) shows an X-ray map and model. In the cryo-EM maps, the ion and water molecules were placed using the automated SWIM procedure (Zhang *et al.*, 2020 ▸). The radial plots at the bottom show the numbers of water molecules and ions at various distances from nearby polar or charged protein atoms (O or N).

Examples of water molecules and ions found in three cryo-EM maps of apoferritin (using the SWIM method) and a previously reported X-ray map are shown in Fig. 8 ▸. When using the SWIM method, waters and ions were placed only if the *Q*-scores of the placed atoms were 0.9 or higher, to ensure that they are indeed well resolved. Fig. 8 ▸ shows three charged residues (Asp and His), around which one ion (green ball) and a few water molecules (red spheres) can be seen. The ion is well resolved in all maps, although the density appears to spread out more at lower resolutions.

Fig. 8 ▸ shows the variability in positions and contour shapes for the water molecules at different resolutions; however, the placed ion appears in a similar position in these maps. Fig. 8 ▸ also shows radial distance plots for the ions and water molecules in each structure from the adjacent protein atoms. A peak number of waters can be seen to occur ∼2.8 Å away from O protein atoms in all structures, although the peak appears sharper at higher resolutions and in the X-ray map. For ions, the X-ray map shows that ion–O distances have a peak at 2.2 Å. In cryo-EM maps, the peak for ions appears to be closer to 2.4 Å. It is interesting that the number of detected water molecules diminishes as the resolution decreases from 1.15 to 1.75 Å, while the number of detected ions does not decrease as dramatically. This may be related to higher scattering from ions due to size and charge. Water-molecule and ion analysis are still new in the field of cryo-EM, and further analyses are certainly needed to better characterize such differences and other potential factors.

## 13. Summary and discussion   

Cryo-EM is continuing to have a large impact in the field of structural biology, providing detailed structures of macromolecules in close-to-native environments and even different functional states. Methods that ensure the validity of the structures and avoid various pitfalls that can lead to wrong interpretations have been discussed here and continue to be of great importance in the field. This started with the use of independent reconstructions enabling ‘gold-standard’ resolution estimation by FSC, and high-resolution noise substitution/phase randomization to ensure that overfitting of noise does not take place in the 3D reconstruction.

Once a map has been obtained, we have reviewed methods for building a model *de novo* in higher resolution maps (typically ∼4 Å and higher) or fitting known atomic structures into lower resolution maps. In the latter, we have discussed the use of geometry restraints to make up for missing information in the map. It has been shown that such restraints can maintain the proper geometry when used properly; however, when the influence of the map is exaggerated overfitting can occur, in which case the map–model scores are improved, but more in the sense that the model better matches noise rather than signal, while model geometry is sacrificed. Geometry-only scores can be calculated with tools such as *MolProbity* (Prisant *et al.*, 2020 ▸), which remain very important in the field. These scores consider valence geometry (bond lengths and angles), backbone dihedrals (Ramachandran statistics), side-chain rotamers, pseudo-dihedrals between adjacent residues (*CaBLAM*) and clashes between nearby atoms.

On the other hand, when refinement and flexible fitting tools more strongly enforce geometry over map–model fit, model-only scores become less meaningful when used to evaluate the resulting model. As a result, map–model scores also remain of great importance to ensure that the model properly reflects the map. Map–model scores include cross-correlation (Joseph *et al.*, 2017 ▸) and statistical scores, for example *EMRinger* (Barad *et al.*, 2015 ▸) or *Z*-scores (Pintilie & Chiu, 2018 ▸). Here, we have focused on a recent score which assesses the resolvability of atomic features in a cryo-EM map at the per-atom and per-residue level: the *Q*-score (Zhang *et al.*, 2020 ▸). Newer analyses including *B* factors and water-molecule/ion detection in cryo-EM maps are also illustrated, and should continue to be an interesting area for further methods development in the context of map validation and interpretation.
